# Supplementary material for: Acaricidal activity of Egyptian crude plant extracts against Haemaphysalis longicornis ticks
Source: PLoS One. 2024 Jul 22;19(7):e0307297. doi: 10.1371/journal.pone.0307297 (PMC11262685; doi:10.1371/journal.pone.0307297)
Supplement: S1 Table — Plants used in this study were collected from the wild survey from the desert roads around Qena Governorate and Luxor governorate and were identified microscopically in South Valley University herbarium, Faculty of Science, South Valley university, Qena, Egypt. Latin binomial names were also provided in the plant identification letter. (PDF) [file pone.0307297.s001.pdf]

S1 Table. Latin binomial name of all crude plant extracts used in this study

Plant Extract	Family	Latin binomial name
Aerva javanica	Amaranthaceae	*Aerva javanica* (Burm.f.) Juss. ex Schult.
Anabasis setifera	Amaranthaceae	*Anabasis setifera* Moq.
Artemisia judaica	Asteraceae	*Artemisia judaica* L.
Carthamus tinctorius	Asteraceae	*Carthamus tinctorius* L.
Citrullus colocynthis.	Cucurbitaceae	*Citrullus colocynthis* (L.) Schrad.
Cleome droserifolia	Cleomaceae	*Cleome droserifolia* (Forssk.) Delile
Forsskaolea tenacissima	Urticaceae	*Forsskaolea tenacissima* L.
Ochradenus baccatus	Resedaceae	*Ochradenus baccatus* Delile
Ocimum basilicum	Lamiaceae	*Ocimum basilicum* L.
Trichodesma africanum	Boraginaceae	*Trichodesma africanum* (L.) Sm.
Origanum majorana	Lamiaceae	*Origanum majorana L.*

Plants used in this study were collected from the wild survey from the desert roads around Qena Governorate and Luxor Governorate and were identified microscopically in South Valley University herbarium, Faculty of Science, South Valley university, Qena, Egypt. Latin binomial names were also provided in the plant identification letter.
