# Supplementary material for: Acaricidal activity of Egyptian crude plant extracts against Haemaphysalis longicornis ticks
Source: PLoS One. 2024 Jul 22;19(7):e0307297. doi: 10.1371/journal.pone.0307297 (PMC11262685; doi:10.1371/journal.pone.0307297)
Supplement: S1 Fig — Map of the plant sample collection sites in Egypt. Plant taxa were collected in a field survey from mid-May 2019 from two sites in the southern part of Egypt in the Qena Governorate desert, and 1 candidate was collected from the Luxor governorate. The collection was performed between 4 AM and 12 PM on 3 successive days. The first site was the Qena-Safaga desert road (X), and the second was the Qena-Sohag desert road (Y). The map was designed by DIVA-GIS 7.5.0 software to illustrate all country roads and governorates; the sample collection sites were approximately determined and are shown in the magnified box as highlighted blue lines on a Google map. (PDF) [file pone.0307297.s002.pdf]

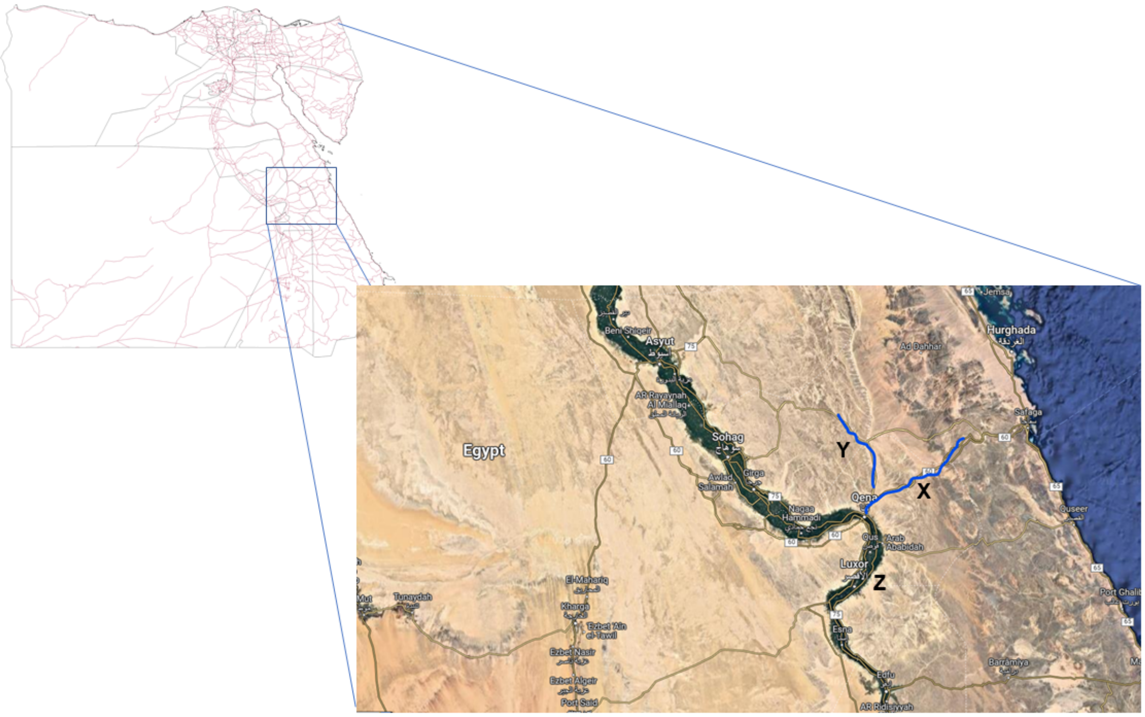

S1 Fig. Sampling map of the plant samples collected in Egypt. Plant taxa were collected during the period from Mid-May, 2019 in a field survey from two sites in the southern part of Egypt in Qena Governorate desert and 1 sample was collected from Luxor governorate. Collection were done between 4 am and 12 PM on successive days. The first site was Qena-Safaga desert road **(X)**, and the second site was Qena-Sohag desert road **(Y)**, Luxor governorate **(Z).** Map designed by DIVA-GIS 7.5.0 software to illustrate all country roads and Governorates; the sample collection sites were approximately determined and are shown in the magnified box as highlighted blue lines on a Google map.
